# Evaluating the effect of temperature on viral survival in plant‐based feed during storage

**DOI:** 10.1111/tbed.14546

**Published:** 2022-04-11

**Authors:** Nicholas Dee, Karyn Havas, Apoorva Shah, Aaron Singrey, Gordon Spronk, Megan Niederwerder, Eric Nelson, Scott Dee

**Affiliations:** ^1^ School of Public Health University of Minnesota Minneapolis Minnesota; ^2^ Pipestone Applied Research Pipestone Veterinary Services Pipestone Minnesota; ^3^ SAM Nutrition Eden Prairie Minnesota; ^4^ Department of Veterinary and Biomedical Sciences South Dakota State University Brookings South Dakota; ^5^ Department of Diagnostic Medicine/Pathobiology, College of Veterinary Medicine Kansas State University Manhattan Kansas

**Keywords:** extended storage, feed, soybean meal, swine, temperature, viral diseases

## Abstract

Viruses of veterinary significance are known to survive for extended periods in plant‐based feed ingredients imported into North America. To reduce the likelihood of virus introduction, high‐risk ingredients, such as oil seed meals, are stored in designated facilities for extended periods under controlled environmental conditions to minimize viral infectivity prior to use in diets. While 30 days has become a standard storage period, the required ambient temperature to inactivate viruses during this time is not known. To address the question, 1‐metric tonne totes of conventional soybean meal were inoculated with PRRSV 144 lineage 1C variant and SVA prior to storage for 30 days at 23.9°C, 15.5°C or 10°C, and feeding to pigs. Virus infectivity was evaluated through detection of viral RNA in oral fluid samples, along with clinical signs. Results indicated that inactivation of both viruses occurred in soy stored at 23.9°C. In contrast, SVA infectivity was observed in soy stored at both 15.5°C and 10°C, while PRRSV 144 L1C variant infectivity was only observed in soy stored at 10°C. These results suggest that a storage period of 30 days and a temperature of 23.9°C may assist in the reduction of the risk of virus contaminated plant‐based feed ingredients, such as soybean meal.

## 1 INTRODUCTION

North America currently imports plant‐based feed ingredients, such as soybean meal, from countries that are endemically infected with viruses of economic and pathologic significance to swine including porcine epidemic diarrhoea virus (PEDV), Seneca virus A (SVA), porcine reproductive and respiratory syndrome virus (PRRSV), classical swine fever virus, pseudorabies virus, foot and mouth disease virus (FMDV) and African swine fever virus (ASFV; Blomme et al., 2022; Patterson, 2022; Patterson et al., 2021). These viruses all survive in conventional soybean meal for 30–37 days, and the *T*½ of ASFV in conventional soybean meal, organic soybean meal and soy oil cake is 9.6, 12.9 and 12.4 days, respectively (Caserta et al., 2022; Dee et al., 2014; Dee et al., 2016; Dee et al., 2018; Stenfeldt et al., 2022; Stoian et al., 2019; Stoian et al., 2020). The North American swine industry has attempted to mitigate this risk through many ways, including mechanical reduction techniques, that is, attempting to decontaminate the feeding system by filling feed lines with corn (flushing) or running repeated batches of feed through the milling equipment (sequencing), after a potentially contaminated batch of feed has run through the mill, heat treatment via pelleting, chemical mitigation and extended storage under controlled environmental conditions (Cochrane et al., 2017; Dee et al., 2020; Gebhardt et al., 2018; Patterson et al., 2019; Schumacher et al., 2018). This latter approach has been applied across North America, with Canada developing a national policy‐based program and the US swine industry adopting a voluntary program known as ‘Responsible Imports’ (Becton et al., 2022; Calvin et al., 2022; Patterson et al., 2019). However, these protocols are not standardized and have been primarily based on mathematical estimates of half‐life, not data derived from controlled challenge studies (Becton et al., 2022; Sundberg, 2020).

To address this limitation, a pilot project was conducted to determine whether 30 days of storage in a climate‐controlled (20°C) environment would reduce the risk of PRRSV 174, PEDV and SVA survival in various feed ingredients, compared to storage in an uncontrolled external environment. This study demonstrated that an environmentally controlled temperature was important for viral inactivation; however, the scale of the study was small (30 g feed allotments), artificial in design (stored in centrifuge tubes), involved only one storage temperature (20°C) and virus infectivity was determined by swine bioassay and not natural feeding (Dee et al., 2021). Therefore, the purpose of this new study was to determine the required ambient temperature to inactivate SVA and PRRSV L1C variant using representative volumes of soybean meal stored in temperature‐controlled environments using industry standards for a 30‐day period, and natural feeding behaviour to determine infectivity. The study was based on the hypothesis that virus survival in feed will be negatively impacted by increasing temperature with the final goal of providing data for the development of industry standards for the management of high‐risk ingredients that could serve as vehicles for the spread of domestic and transboundary diseases.

## 2 MATERIALS AND METHODS

### 2.1 Ethical statement

Animals in this study were managed in accordance with the institutional animal care and use guidelines observed by the investigators’ ethical review board, Pipestone Applied Research IACUC, trial number 2021–13.

### 2.2 Experimental facilities

The study utilized three facilities: a commercial warehouse for feed tote preparation and inoculation, a temperature‐controlled trailer for the 30‐day storage of totes and the Pipestone Research biosafety level 2 (BSL‐2) facility for the assessment of virus infectivity in pigs. At the warehouse, a total of eighteen 1‐tonne totes of conventional soybean meal were prepared. As previously defined, conventional soybean meal contained a low fat (1–2%) and high protein (46–47%) content. This ingredient was added to new polypropylene bags each with a capacity of 1.74 m^3^ (National Bulk Bag, Champlin, MN USA), resulting in the 18 totes to be used in the study. This number of totes was based on six totes per temperatures to be tested (23.9°C, 15.5°C or 10°C) and the six available rooms in the BSL‐2 facility. As the experimental unit was the room of pigs, a sample size of six rooms per temperature was used and could detect a 75% difference in infection rates with a 95% confidence and 80% power.

### 2.3 Sample preparation and tote inoculation

Viruses selected for inoculation included PRRSV‐144 L1C variant and SVA, based on the stability of SVA in soybean meal (Dee et al., 2018, Caserta et al., 2022) and the recent emergence of the highly pathogenic PRRSV‐144 L1C variant and subsequent industry concern of potential transmission through feed (Trevisan et al., 2021). To simulate a ‘hot spot’ model of feed contamination, 10 ml ice cubes containing a mixture of PRRSV 144 L1C variant and SVA, each at a total dose of 1 × 10^5^ TCID_50_ per virus, were prepared using a previously published approach (Dee et al., 2022). Stock viruses originated from an accredited laboratory, where they were purified, propagated and titred using standard virological techniques. Each virus was diluted in 30 ml of minimum essential medium (MEM, Sigma‐Aldrich, St. Louis, MO, USA). Ice cubes were prepared by freezing 10 ml aliquots of the mixture in 50 ml conical centrifuge tubes (Corning Inc. Corning, NY, USA) at −80°C. The 18 totes were divided into three groups of six, according to the three temperatures to be tested. The first set of totes was inoculated and stored at 23.9°C. To inoculate totes, a solid metal rod (1.2 m in length and 4.45 cm in diameter) was inserted into the lumen of a PVC pipe (1.2 m in length and 5.08 cm in diameter). The combined rod and pipe ‘inoculation instrument’ was manually forced into the middle of each tote so as only 0.35 m of the instrument was not covered by feed material. Once the instrument was in the proper place, the internal metal rod was removed, providing a clear path for the ice cube to travel into the interior of the tote, unimpeded by the presence of meal. Following removal of the rod, the 10 ml ice cube was dropped into the PVC tube, the PVC tube was removed, resulting in the cube being buried by the meal and creation of the ‘hot spot’ centralized in the tote.

### 2.4 Storage procedure

Immediately following inoculation, each set of six totes were placed into temperature‐controlled trailer and stored for 30 days at the designated temperature assigned to each replicate. The trailer was a 2017 14.6 m Hyundai Thermotech Refrigerated Trailer with a Thermoking reefer unit (Sumrall Truck and Trailer Service, Hammond, LA, USA). The 23.9°C replicate was conducted first, followed by the 15.5°C replicate, then the 10°C replicate. These three temperatures were selected based on current implementation across the US swine industry. The trailer was cleaned and disinfected and allowed to sit empty for 30 days between replicates.

### 2.5 Virus infectivity assessment

Following completion of the designated 30‐day storage period, each set of six totes was transported to the BSL‐2 facility in the refrigerated trailer. This facility had six rooms, each with its own feed bin, allowing for an individual tote to be allocated to a specific bin and be fed to a specific group of pigs. These rooms were organized into separate airspaces using filtration of incoming and outgoing air, with separate shower in/out and Danish entry protocols per room, along with the use of separate clothing, footwear, disposable gloves and equipment per room to prevent room‐to‐room cross‐contamination. During each replicate, each room in the facility was stocked with 30 six‐month‐old finishing pigs, originating from a PRRSV and SVA negative farm. This farm had been documented free of both diseases thorough monthly clinical observation by the attending veterinarian, along with sampling of suckling pigs and replacement gilts over several years. Offspring from these farms had been monitored as well with no history of infection in animals tested upon arrival across numerous wean to finish sites. This number and size of pigs was chosen based on an estimated average daily feed intake of 2.3 kg/pig. At this rate of consumption, it was estimated that it would require approximately 14 days to consume the entire amount of soybean meal placed in the feed bin. Upon disappearance of the soybean meal, the animals were fed a balanced diet to offset any nutritional deficiencies provided by the soy‐only diet. Prior to the experiment, this approach was discussed with the Pipestone IACUC and the Pipestone Nutrition team to anticipate and mange any concerns that could arise from feeding straight soybean meal for 14 days. No concerns were noted.

During the 30‐day replicate, all six rooms of pigs were assessed for evidence of SVA and PRRSV 144 L1C infection using a pen‐based sampling method through the collection of oral fluids (one rope per pen, six ropes per room) at day 0 (arrival), 14 and 30 of each replicate. As this study utilized natural feeding behaviour and not purposeful animal inoculation at a specific point in time, we developed a sampling protocol to evaluate infection over the course of the feeding period. This approach was particularly important, since the transfer of each tote into its designated bin could result in random mixing of the hot spot throughout the tonnage, resulting in the potential for a high degree of variability in viral exposure as the pigs consumed the feed. Evidence of viral infection was monitored using a previously published approach (Dee et al., 2020; Dee et al., 2022) that included the collection of oral fluid samples that were tested for the presence of PRRSV RNA and SVA RNA by polymerase chain reaction at the South Dakota State University Animal Disease Research Diagnostic Laboratory. In addition, daily observations were conducted, looking for clinical signs of SVA infection (vesicles and lameness) and signs of PRRSV 144 L1C variant infection (pyrexia, hyperaemia, dyspnoea and weight loss). Selected cases of mortality were necropsied, lymphoid tissues collected (tonsil, lymph nodes and spleen) and tested by PCR for the presence of SVA and PRRSV RNA.

### 2.6 Environmental monitoring during storage

During each replicate, two temperature and relative humidity (% RH) data loggers (RC‐51H waterproof USB temperature data logger, Elitech, San Jose, CA, USA) were placed within the trailer to collect environmental data within the trailer during the storage period. Each logger was placed 11.5 cm from the floor, on the wall surface in adjacent corners. One was placed in the rear end of the trailer on the right side, and one was placed in the front of the trailer on the left side. Placement of loggers was consistent for all three replicates.

### 2.7 Statistical analysis

Due to small sample sizes, a two‐sided Fisher's exact test was used to assess for the significance of the association between tote holding temperature and room‐level infection rates overall. A Fisher's exact two‐sided test was used when the association was stratified by pathogen, again due to the small sample sizes. The level of significance used for the omnibus comparison was 0.1 and the pairwise temperature comparisons *p* values were evaluated against levels of significance calculated using the Benjamini–Hochberg method with a false discovery rate equal to the level of significance (.1) as described (Benjamini & Hochberg, 1995). This latter procedure was employed to statistically enhance the power of the study while conducting multiple comparisons as opposed to traditional, more conservative adjustments such as the Bonferroni correction. The Benjamini–Hochberg method ranked the *p* values and generated different ‘cut‐off values’ based on the false discovery rate and the rank number of the comparison being made. STATA version 16.1 IC statistical software (Stata Corporation, College Station Texas) was used with data stored in Microsoft Excel version 16.56 spreadsheets (Microsoft Corporation, Redmond, WA, USA).

## 3 RESULTS

### 3.1 Clinical and diagnostic observations by replicate

All oral fluid samples collected from all pens across the three replicates were PCR negative for SVA and PRRSV RNA on day 0 (arrival of pigs). During the 23.9°C replicate, oral fluid samples collected at 14 and 30 days were PCR negative for both viruses, and clinical signs were not observed. In the 15.5°C replicate, SVA RNA was detected in oral fluid samples, with Ct values ranging from 25.8 to 36.1, and vesicles on the feet and snout of multiple animals in each room along with severe lameness were observed in five of the six rooms. No PRRSV‐positive oral fluids were detected, and no PRRSV‐related clinical signs were observed. In support of these data, SVA RNA was detected in tissue samples from selected clinically affected animals that had died during the study period (Ct = 25.8–29.4). In the 10°C replicate, SVA RNA in oral fluid samples were detected (Ct = 29.4–37.1) and severe clinical signs of SVA were observed in four of six rooms. In addition, PRRSV RNA was detected in oral fluid samples (Ct = 24.9–36.7), along with observation of severe clinical signs of PRRSV in four of the six rooms. In support of these data, SVA RNA (Ct = 31.1–36.1) and PRRSV 144 L1C variant RNA (Ct = 26.4–33.5) were detected in tissue samples from selected clinically affected animals that had died during the study period.

### 3.2 Data analysis

There was an overall association between tote holding temperature and rate of infection with SVA or PRRSV with a *p* = .005 (Table 1). Overall, there was a significant (*p* = .015) association between tote holding temperatures (23.9°C vs. 15.5°C and 23.9°C vs. 10°C) and detection of either SVA or PRRSV (Table 1), with feed held at 23.9° not having any infected rooms detected. Further analysis revealed an association between disease presence and temperature of storage when comparing 10°C to 23.9°C and 15.5°C to 23.9°C, as both *p* values were below the sequential Benjamini–Hochberg statistic cut‐off values, and there was no difference between 10°C and 15.5°C. When the data were stratified by pathogen, there were still significant differences in the holding temperatures and the infectivity of the soy for both pathogens overall (*p* value_SVA _= .025; *p* value_PRRSV _= .015) (Table 2). Furthermore, there were different patterns of significant associations that emerged between holding temperatures based on the pathogen. For SVA, the rate of infection did not reach zero for all rooms until the soy was held at 23.9°C. The 23.9°C holding temperature was significantly different from the 15.5°C and 10°C, while the latter two were not different from one another. For PRRSV 144 L1C variant, all the rooms remained uninfected when fed soy held at 15.5°C and 23.9°C. In contrast, 66.7% of the rooms were infected when fed soy held at 10°C. Finally, since all rooms tested negative at 15.5°C and 23.9°C, a comparison was not possible.

**TABLE 1:** Detection of porcine reproductive and respiratory syndrome virus or Senecavirus A in rooms of pigs exposed via contaminated feed‐by‐feed tote storage temperature

	Disease positive # (%)	Sample size #	*p* Value	Benjamini–Hochberg statistic
Overall			.005	
23.9°C	0 (0%)	6		
15.5°C	5 (83.3%)	6		
10.0°C	5 (83.3%)	6		
10.0°C vs. 15.5°C	–	–	1	.1
10.0°C vs. 23.9°C	–	–	.015	.033
15.5°C vs. 23.9°C	–	–	.015	.067

**TABLE 2:** Detection of infected pigs by pathogen in rooms of pigs exposed via contaminated feed‐by‐feed tote storage temperature

	PRRS positive # (%)	Sample size #	*p* Value	Benjamini–Hochberg statistic	SVA positive # (%)	Sample size #	*p* Value	Benjamini–Hochberg statistic
Overall			.015				.025	
23.9°C	0 (0%)	6			0 (0%)	6	–	–
15.5°C	0 (0%)	6			5 (83.3%)	6	–	–
10.0°C	4 (66.7%)	6			4 (66.7%)	6	–	–
10.0°C vs. 15.5°C	–	–	.061	.05	–	–	1	.1
10.0°C vs. 23.9°C	–	–	.061	.1	–	–	.061	.067
15.5°C vs. 23.9°C	–	–	Not tested		–	–	.015	.033

### 3.3 Temperature and % relative humidity data

Over the course of the 30‐day period at a storage temperature of 23.9°C, the mean temperature across both loggers was 22.8°C with a mean RH of 62.4%. At a storage temperature of 15.5°C, the mean temperature across both loggers was 15.3°C with a mean RH of 63.4%. Finally, at a storage temperature of 10.0°C, the mean temperature across both loggers was 9.5°C with a mean RH of 27.5%.

## 4 DISCUSSION

The purpose of this study was to determine the required ambient temperature to inactivate two significant viral pathogens of pigs during the storage of soybean meal for a 30‐day period. Under the conditions of this study, we learned that holding feed at higher temperatures, that is, 23.9°C, significantly reduced infectivity of SVA and PRRSV L1C variant. We also learned that SVA demonstrated greater survivability than PRRSV, as evidenced by five of the six rooms developing disease when fed contaminated soy held at 15.5°C, while the PRRSV infectivity was neutralized when contaminated soy was held at 15.5°C, but not 10.0°C. Since we did not collect feed samples and measure viral load over time, we cannot conclude that the SVA results are solely due to it greater ability to survive in feed; however, this is a logical hypothesis based on historical data and should be investigated further.

The strengths of this study were its practical approach and the use of a rigorous experimental design to answer the specific research question. The diagnostic testing protocol used in the study effectively proved the presence or absence of infection at the level of the room through the detection of viral RNA in a population‐based sampling plan. The accuracy of the results was maximized as an oral fluid sample was collected from each of the six pens in each of the six room. Additionally, each pen only contained five pigs; therefore, accessibility to ropes in the pens was not compromised. As room was the experimental unit, we were only concerned if the overall population in the room was determined to be positive, not how many pens eventually became infected. Specifically, once one or more pens in a room were positive, the room was determined to be positive. Therefore, we did not conduct routine serological monitoring of individual animals. Nor did we place dividers between pens to limit viral spread, as we considered the spread of either virus across the pens as evidence that infection had occurred in the room. In addition, the detection of the viruses in systemic tissue samples proved additional evidence that the animals tested were truly infected.

Another strength was the use of SVA, a virus known to be very stable in feed, as well as a validated surrogate for FMDV, in combination with an ingredient (conventional soybean meal) known to be very protective to multiple viruses, to generate a ‘worst case scenario’ to test the efficacy of the protocol under BSL‐2 conditions. As mentioned, the outcomes of the study are important, since previous storage periods for feed were based only on mathematical half‐life calculations, not controlled studies using live pathogens and representative conditions. Also, maintaining a consistent 23.9°C temperature over time appeared to be important, as previous studies have demonstrated the ability of these viruses to survive for greater periods in soy (Caserta et al., 2022; Dee et al., 2018). Finally, an additional strength of the study was the high quality of biosecurity available at the BSL‐2 facility. This facility has been in use for over 2 years and cross‐contamination between rooms has not been observed to occur. An example of this level of rigour is the fact that in the 10°C replicate, individual virus infection (PRRSV or SVA only) was observed in certain rooms.

In contrast, the major limitation to this study is the sample size. The sample size used could only detect ≥75% difference between holding temperatures with 95% confidence and with 80% error and ≥65% difference at a 90% confidence. When making multiple comparisons, it is appropriate to adjust the level of significance, and in doing this, it further limited detection despite using a methodology that tries to conserve power. As a result, the 66.7% difference in PRRSV infection rates of rooms fed feed held at 10°C, versus 15.5°C and 23.9°C, was not statistically significant. Nonetheless, the experiment was repeated for two separate pathogens and resulted in a similar conclusion that storing feed at higher temperatures reduces pathogen infectivity. Another consideration that needs discussion is the potential for false negative results, due to the subclinical nature of these diseases in growing pigs. While we did not routinely necropsy healthy animals, there were several sudden death cases that were investigated and intestinal torsions were observed. As described, lymphoid tissues were collected, and all samples were PCR negative for both viruses. Unfortunately, due to the BSL‐2 qualifications of the research facility, we could not use ASFV in the study; we did use SVA, a virus previously shown to be even more stable in feed (Dee et al., 2018). In addition, a limitation of the method of inoculation of soy, while potentially representative of what may happen in the field, does not insure that all animals in each treatment group will become challenged. However, as the room was the experimental unit, it was not required that all animals or pens become infected.

Finally, additional limitations include a lack of understanding of the infectious dose of either virus, the potential effect of additional time and environmental temperature on the viruses during storage in the feed bin at the farm. In addition, we observed a lower % RH during the 10^0^ C storage period, although the significance of this observation is unknown. We also did not attempt to collect feed samples and measure virus reduction over time, due to the challenges of sampling large volumes of grain which were inoculated with a method purposely designed to create a very limited point of contamination (‘hot spot’). One could argue whether this method of contamination is realistic and question the effects of the liquid component on viral survival. While these points are legitimate criticisms, hot spot contamination has been described for alfatoxin (FDA CVM, 2019) and we purposely tried to reduce the amount of liquid in the challenge as compared to other attempts. We also did not want to enter the storage units during the respective 30‐day periods to avoid artificially modifying the temperature following entry of ambient air. Finally, it must be recognized that these results are based on a ‘worst case scenario’ where the experimental design used a highly supportive feed matrix, and the results may not be applicable across all types of feed and feed ingredients.

In closing, although we used a ‘worst case scenario’ example, we now have, for the first time, important evidence to advise farmers, feed mill operators, federal officials, regulatory and practicing veterinarians and feed industry leadership on how long and at what temperature to store feed and feed ingredients, to minimize risk. Hopefully, this information will enhance the application and efficacy of Responsible Imports protocols as we collectively work to manage the global risk of feed.

## FUNDING

Resources for this study were provided by the Swine Health Information Center, SAM Nutrition and Pipestone Applied Research.

## CONFLICT OF INTEREST

The authors declare no conflict of interest.

## Data Availability

All data from this study have been disclosed.
